# Comprehensive analysis of keloid super-enhancer networks reveals FOXP1-mediated anti-senescence mechanisms in fibrosis

**DOI:** 10.1186/s11658-025-00763-1

**Published:** 2025-07-23

**Authors:** Hao Yang, Dongming Lv, Xiaohui Li, Yongfei Chen, Hailin Xu, Honglin Wu, Zhiyong Wang, Xiaoling Cao, Bing Tang, Wuguo Deng, Jiayuan Zhu, Zhicheng Hu

**Affiliations:** 1 Department of Burn and Wound Repair, Sun Yat-Sen University First Affiliated Hospital, Guangzhou, 510080 China; 2 State Key Laboratory of Oncology in South China, Collaborative Innovation Center of Cancer Medicine, Sun Yat-Sen University Cancer Center, Guangzhou, 510060 China; 3 Guangdong Provincial Hospital of Integrated Traditional Chinese and Western Medicine, Foshan, 528200 China; 4 Guangdong Provincial Dermatology Hospital, Department of Dermatology, Dermatology Hospital of Southern Medical University, Guangzhou, 510091 China; 5 Department of Joint Surgery, The Third Affiliated Hospital of Guangzhou Medical University, Guangzhou, 510150 China

**Keywords:** Integrative omics analysis, Keloid, Super-enhancers (SEs), Transcription factors (TFs), Anti-senescence, Fibrosis

## Abstract

There is a significant gap in multi-omics studies on keloids, especially concerning the interaction between fibroblasts and super-enhancers (SEs). Identifying novel biomarkers within the epigenetic landscape could greatly improve keloid management. In this study, we investigated gene expression at both transcriptional and translational levels to identify potential biomarkers and employed CUT&Tag technology to validate SE-associated genes and upstream transcription factors (TFs). Through integrated analyses of transcriptomics and proteomics, 10 hub genes that associated with ECM, immune, and metabolic pathways were found. Given the crucial role of fibroblasts in keloid pathogenesis, we further identified five SE-associated genes (SERPINH1 SE, MMP14 SE, COL5A1 SE, COL16A1 SE, and SPARC SE) that exhibit characteristic upregulation in keloids. Analysis of upstream TFs and core transcription regulatory circuitry (CRC) revealed potential master TFs (FOSL2, BACH2, and FOXP1), with FOXP1 emerging as the core TF likely driving pro-fibrotic development through its anti-senescence function. In summary, we anticipate that the outcomes of the integrative omics analysis will facilitate further investigation into the underlying molecular mechanisms of keloid formation and lead to novel strategies for its prevention and management. Specifically inhibiting the anti-senescence function of FOXP1 brings new promise for the treatment of fibrosis-related diseases.

**Supplementary Information:**

The online version contains supplementary material available at 10.1186/s11658-025-00763-1.

## Introduction

Wound healing is a crucial process for restoring the skin’s barrier function, and scar healing, as a type of wound healing, typically does not extend beyond the boundaries of the wound [1, 2]. As a unique pathological healing process, keloid disease is classified as a fibroproliferative skin disorder characterized by excessive deposition of extracellular matrix (ECM) and shares many similarities with tumors [3, 4]. Keloids primarily occur on the chest, back, earlobes, and other areas with clinical manifestations of invasive growth beyond the wound site. Furthermore, they do not gradually subside and are often accompanied by local itching, pain, and limited range of motion that significantly impact both the function and appearance of the affected area as well as patients’ quality of life [5, 6]. Currently, there are various clinical interventions available for keloids, including surgical excision, radiotherapy, pressure therapy, pharmacotherapy, and intralesional corticosteroid injection [7]. However, these treatment regimens have high recurrence rates and poor efficacy with unsatisfactory treatment outcomes [8]. Therefore, delving into the molecular mechanisms of keloids can enhance our comprehension of their pathogenesis and facilitate the development of novel diagnostic and therapeutic strategies for patients with keloids.

Previous studies have investigated transcriptomic alterations in keloid tissue using bulk RNA-seq or microarray analysis [9–11]. With the aid of bioinformatics, it enables the secondary utilization of sequencing data, fully exploiting the information provided by such data and identifying biomarkers for various diseases [12]. These studies underscore the utility of genetic and bioinformatics analyses as valuable tools for exploring molecular mechanisms and identifying potential diagnostic and therapeutic targets for human skin diseases. However, previous investigations have not endeavored to correlate transcriptional changes with alterations in protein levels, which may be more pertinent to biological phenotypes. Therefore, the integration and analysis of comprehensive characteristics of keloid gene expression in combination with multiple recombinomics through the progress of proteomic and transcriptomic studies offer novel insights into understanding the mechanism underlying keloid pathogenesis. Furthermore, to our knowledge, no systematic study based on multiomics has been conducted to investigate regulatory changes associated with keloids.

Super-enhancers (SEs) consist of a cluster of closely activated enhancer elements that are bound by key transcription factors (TFs) and mediating coactivator complexes, which together maintain cell type-specific properties and determine the cell’s fate [13]. As a pivotal regulatory center that integrates environmental cues and regulates genome expression, this plays an indispensable role in controlling diverse biological processes during mammalian development [14]. SEs have the ability to regulate gene expression that is closely associated with cellular identity. Typically, these enhancers are located within a genomic distance of 12.5 kb [15]. SEs are strongly associated with the expression of pathogenic genes in a variety of diseases, including tumorigenesis, Alzheimer’s disease, diabetes, and autoimmune disorders [14]. Relevant research has demonstrated that SEs play a crucial role in maintaining tumor characteristics and are significantly associated with higher clinical stage and pathological grade of tumors [16]. The maintenance of high transcriptional output in cancer cells is believed to be driven by SEs, indicating that these cells can engage in regulatory networks controlled by such enhancers [17]. Although significant advancements have been achieved in the current investigation of SEs and their associated protein-coding genes [18–20], investigation into the expression profile of SEs in keloids remains inadequate.

**Fig. 1:**
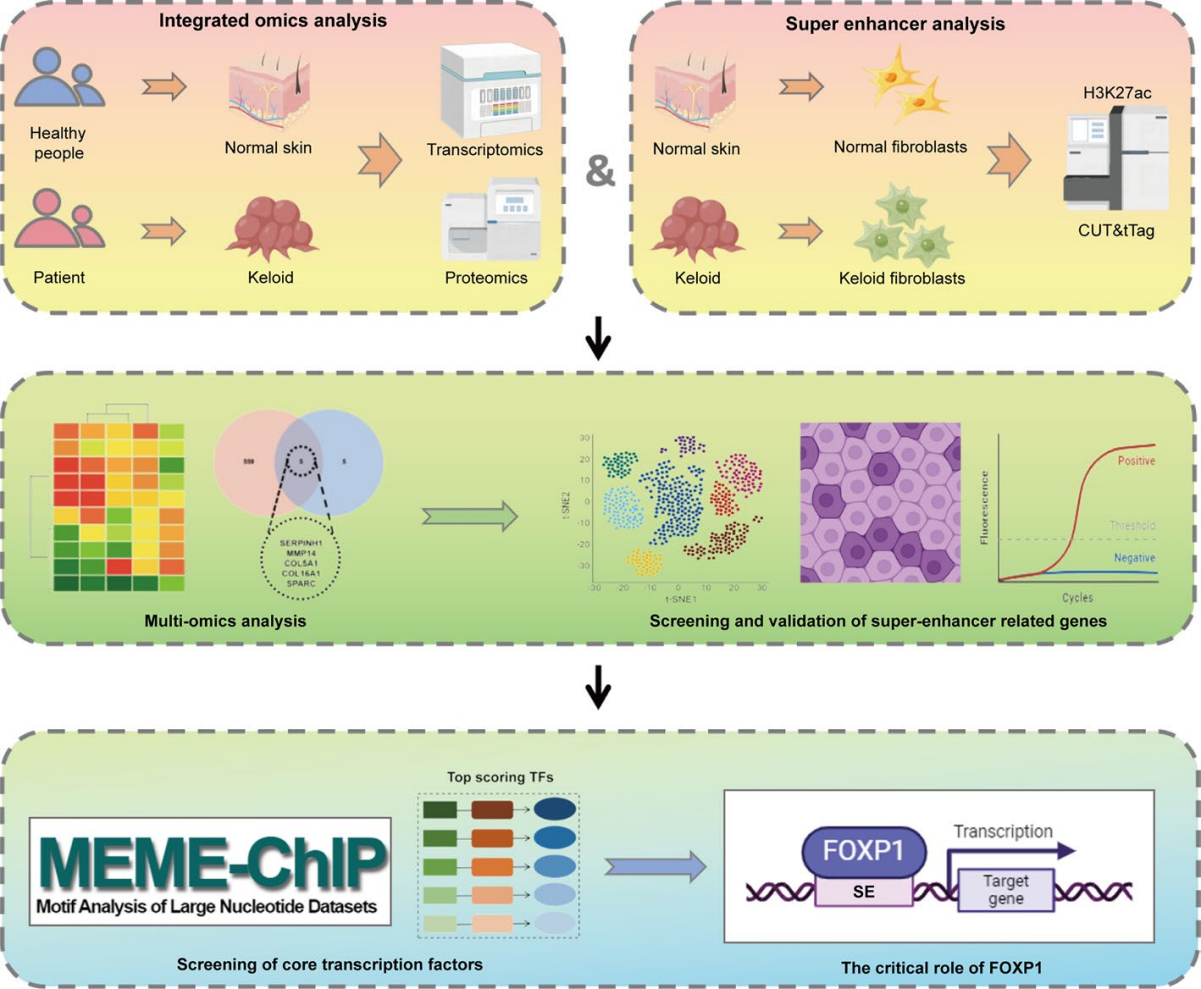
The flowchart of the study

To gain deeper insights into keloid pathology, we conducted an integrated analysis of transcriptomics and proteomics. This approach enabled us to identify key molecular features and signaling pathways, uncovering the characteristic pathological processes underlying keloid formation. In addition, we investigate the landscape of SE-associated genes in keloid fibroblasts and explore the role of FOXP1 as a core transcriptional regulator in this context. By multi-omics analyses, we aim to elucidate the molecular mechanisms driving keloid pathogenesis and identify novel biomarkers and therapeutic targets. Our findings suggest that FOXP1 may not only serve as a critical regulator of fibrosis but also possess an anti-senescence function that could offer new avenues for the treatment of fibrotic diseases (Fig. 1).

## Methods

### Clinical sample collection and sequencing analysis

The study population comprised keloid tissue from three patients undergoing keloid surgery and healthy skin tissue from three patients undergoing benign mass resection at the First Affiliated Hospital of Sun Yat-sen University, Guangdong Province, China. Initially, transcriptome and proteome sequencing analyses were conducted on the aforementioned six tissues. Sequencing processing can be referred to previous literature reports [21]. Subsequently, an additional set of three keloid tissues and three healthy skin tissues were procured for validation experiments. This study was granted approval by the Ethics Committee of the First Affiliated Hospital of Sun Yat-sen University (no. 2021858), and each patient provided written informed consent. For comprehensive information regarding patients and specimen usage in each trial, refer to Supplementary File 1. Transcriptome analysis and proteome analysis were assisted by Gene Denovo Biotechnology Co (Guangzhou, China).

### Differentially expressed genes (DEGs) and differentially expressed proteins (DEPs)

The differential expression analysis of RNAs was performed using DESeq2 [22] software to compare two distinct groups, while edgeR [23] was utilized for comparing two samples. Differentially expressed genes/transcripts were identified on the basis of a false discovery rate (FDR) parameter below 0.05 and an absolute fold change ≥ 2. Protein abundance was determined using Student’s *t*-test, and normalization was achieved by calculating the average abundance of all peptides. Median values were utilized for further averaging. Proteins exhibiting fold changes greater than 1.2 and at least one unique peptide with a *P*-value less than 0.05 were identified as differentially expressed.

### Pathway enrichment analysis

Genes typically interact with each other to perform specific biological functions. Pathway-based analysis facilitates the understanding of gene functions, and KEGG [24] is a primary public database for pathways. Through pathway enrichment analysis, significantly enriched metabolic or signal transduction pathways can be identified among DEGs when compared with the entire genome background.

### GO enrichment analysis

GO enrichment analysis was conducted to identify significantly enriched GO terms in DEPs compared with the genomic background. The DEPs were then filtered based on their corresponding biological functions. Firstly, all DEPs were mapped to the Gene Ontology database (http://www.geneontology.org/), and the number of genes for each term was calculated. Hypergeometric tests were used to determine the significantly enriched GO terms in DEPs compared with the genomic context.

### Correlation analyses

The genes and proteins, as well as the differentially expressed ones identified in both transcriptome and proteome analyses, were tallied separately. A Venn diagram was constructed on the basis of these data, while a four-quadrant map analysis was generated to illustrate the correlation between these molecular entities. The R programming language (version 3.5.1) was utilized for conducting this analysis.

### Protein interaction

The protein–protein interaction (PPI) network was obtained from the STRING database and reconstructed using Cytoscape software [25, 26]. The degree of connectivity for each node in the network was calculated. Subsequently, the molecular complex detection (MCODE) algorithm was employed to identify clusters on the basis of topology that corresponded to densely connected regions.

### Data processing

We downloaded one keloid single-cell RNA sequencing (scRNA-seq) dataset (GSE163973) from the GEO database. After standardization, three keloid tissue samples from GSE163973 were finally analyzed. We selected cells with more than 300 expressed genes, fewer than 5500 expressed genes in total, and less than 10% mitochondrial genes. A total of 25,104 cells were retained. Then, 3516 hypervariable genes were selected for analysis. Using principal component analysis, cell clusters can be obtained, which are then shown as a uniform manifold approximation and projection (UMAP) plot. We then annotated the generated clusters, resulting in five final clusters. The analysis of single-cell sequencing data was facilitated by the use of OmniAnalyzer Pro (Beijing, China) [27]. 

### Fibroblasts were extracted and cultured

Primary keloid fibroblasts and normal human skin fibroblasts were isolated from keloid and normal skin samples, respectively, using established protocols [28, 29]. The tissues underwent thorough washing with PBS to remove the epidermis and subcutaneous adipose tissue before being cut into 1–3-mm^3^ pieces. Following digestion, the resulting suspension was filtered and centrifuged at 1500 rpm for 5 min. Both keloid fibroblast and normal fibroblast cultures were maintained in a CO_2_ incubator and cultured in Dulbecco’s modified Eagle’s medium (DMEM) supplemented with 10% fetal bovine serum (FBS) and 1% antibiotic–antimycotic (100X). All experiments were conducted using cells between passage 1 and 5 (P1-P5). No cells beyond passage 5 were used to prevent senescence or phenotypic alterations [30]. The cleavage under targets and tagmentation (CUT&Tag) experiments (H3K27ac or FOXP1 tagged) were assisted by Epibiotek Co (Guangzhou, China).

### Identification and analysis of SEs

Cutadapt (v2.5) was employed for adapter trimming and sequence filtering, while bowtie2 (v2.3.5.1) with default parameters was utilized to align sequencing reads to the human genome build hg38. The regions of H3K27ac enrichment were determined using MACS2 (v2.1.2), and the resulting MACS peaks of H3K27ac served as constituent enhancers for identification of SEs. Enhancers were identified and classified as SEs using the ROSE algorithm (https://bitbucket.org/young_computation/rose), following established protocols [13]. For enhancer annotation, we employed the homer utility annotatePeaks.pl (v4.10.4) to associate peaks with their proximal genes.

### Real-time quantitative reverse transcription polymerase chain reaction (RT-qPCR)

Keloid and normal skin tissues were surgically excised, immediately snap-frozen in dry ice, and subsequently stored at −80 ℃. Total RNA was isolated from rapidly frozen tissues using TRIzol reagent (Invitrogen), followed by reverse transcription according to the manufacturer’s instructions (TaKaRa™) and real-time polymerase chain reaction using TaKaRa SYBR Premix Ex Taq II (Perfect Real Time). GAPDH was utilized as an internal control, and the primer sequences are provided in Supplementary File 2.

### Immunohistochemical (IHC) staining

Tissue sections obtained from patients were deparaffinized using xylene, and antigen retrieval was performed by subjecting the samples to full-power pressure cooking for 5 min in 10 mM sodium citrate buffer (pH 8.0). The sections were treated with 3% hydrogen peroxide at room temperature for 25 min to inhibit endogenous peroxidase activity, followed by blocking of endogenous antigens using 3% bovine serum albumin (BSA). Subsequently, the sections underwent incubation with both primary and secondary antibodies. The antibodies are provided in Supplementary File 3. The sections were visualized using 3,3′-diaminobenzidine (DAB) chromogenic solution and counterstained with hematoxylin before mounting.

### Computational reconstruction of core transcription regulatory circuitry (CRC)

The coltron python package (https://pypi.org/project/coltron/) was employed to conduct analysis of SE-mediated CRC in accordance with a previously reported methodology [31]. Briefly, the interaction network of all TFs assigned to specific genes was constructed on the basis of their inward and outward degrees that quantify connectivity among all nodes. The inward degree of a TF node represented the total number of other TF nodes with motifs enriched within the proximal regulatory regions of the gene associated with that TF. Conversely, the outward degree of a TF node represented the total number of target genes containing a proximal regulatory region with motifs recognized by that particular TF. In this regulatory model, the interconnected autoregulatory networks (clique) composed of distinct combinations of TFs are defined as CRC. Within the CRC TF cliques, each member binds to its own specific enhancer and those of other TFs in the circuit, thereby co-driving an extended catalog of enhancers.

### Western blot analysis

The proteins were extracted from the cells and quantified using a bicinchoninic acid (BCA) kit. A total of 30 µg protein per sample was utilized for western blot analysis. The proteins were separated on a 10% SDS–PAGE gel and subsequently transferred onto polyvinylidene fluoride (PVDF) membranes. Following blocking with 5% BSA at room temperature for 1 h, the blots were incubated overnight at 4 °C with specific primary antibodies. On the following day, the blots were incubated with secondary antibodies for another hour. Finally, image acquisition was performed using a MiniChemi chemiluminescence imager. The antibodies are provided in Supplementary File 3. The relevant images can be found in Supplementary File 9. 

### Collagen gel contraction, migration, and invasion assays

The keloid fibroblasts were combined with rat tail collagen (5 mg/ml) and 10 × DMEM, followed by incubation in a 24-well plate at 37 °C for 30 min. Subsequently, the resulting collagen gels were photographed after either 24 or 48 h. The difference in gel area was then calculated. For cell migration and invasion assays, the experimental procedure was conducted according to previously described methods [32].

### Senescence-associated β-galactosidase (SA-β-gal) staining

According to the protocol of a SA-β-gal staining kit (Beyotime Biotechnology), cells were washed with PBS and incubated in stationary liquid for 15 min at room temperature. After three washes with PBS for 3 min each, cells were then incubated in the staining working solution at 37 °C without CO_2_ for 14 h. The following day, the staining working solution was discarded and cells were washed three times with PBS. Finally, the samples were examined under a microscope.

### Immunofluorescence (IF)

Cells were washed three times with PBS, fixed with 4% paraformaldehyde for 20 min, and permeabilized with 0.5% Triton X-100 for 20 min. After blocking with 5% goat serum for 40 min, the cells were incubated overnight at 4 °C with collagen I and III antibodies. They were then incubated for 60 min at room temperature with Cy3 or CoraLite 488-conjugated secondary antibodies, followed by DAPI nuclear counterstaining. Finally, the samples were washed, mounted with an anti-fluorescence quencher, and observed under a fluorescence microscope. The antibodies are provided in Supplementary File 3.

### Statistical analysis

Data analysis and figure generation were conducted using GraphPad Prism version 8.0. Results are expressed as the mean ± standard error of the mean (SEM). Intergroup comparisons were analyzed using either a *t*-test or one-way analysis of variance (ANOVA). Each experiment was independently repeated three times, and a significance level of *P* < 0.05 was considered statistically significant for all tests. 

## Results

### Transcriptomic and proteomic analysis of keloid

**Fig. 2:**
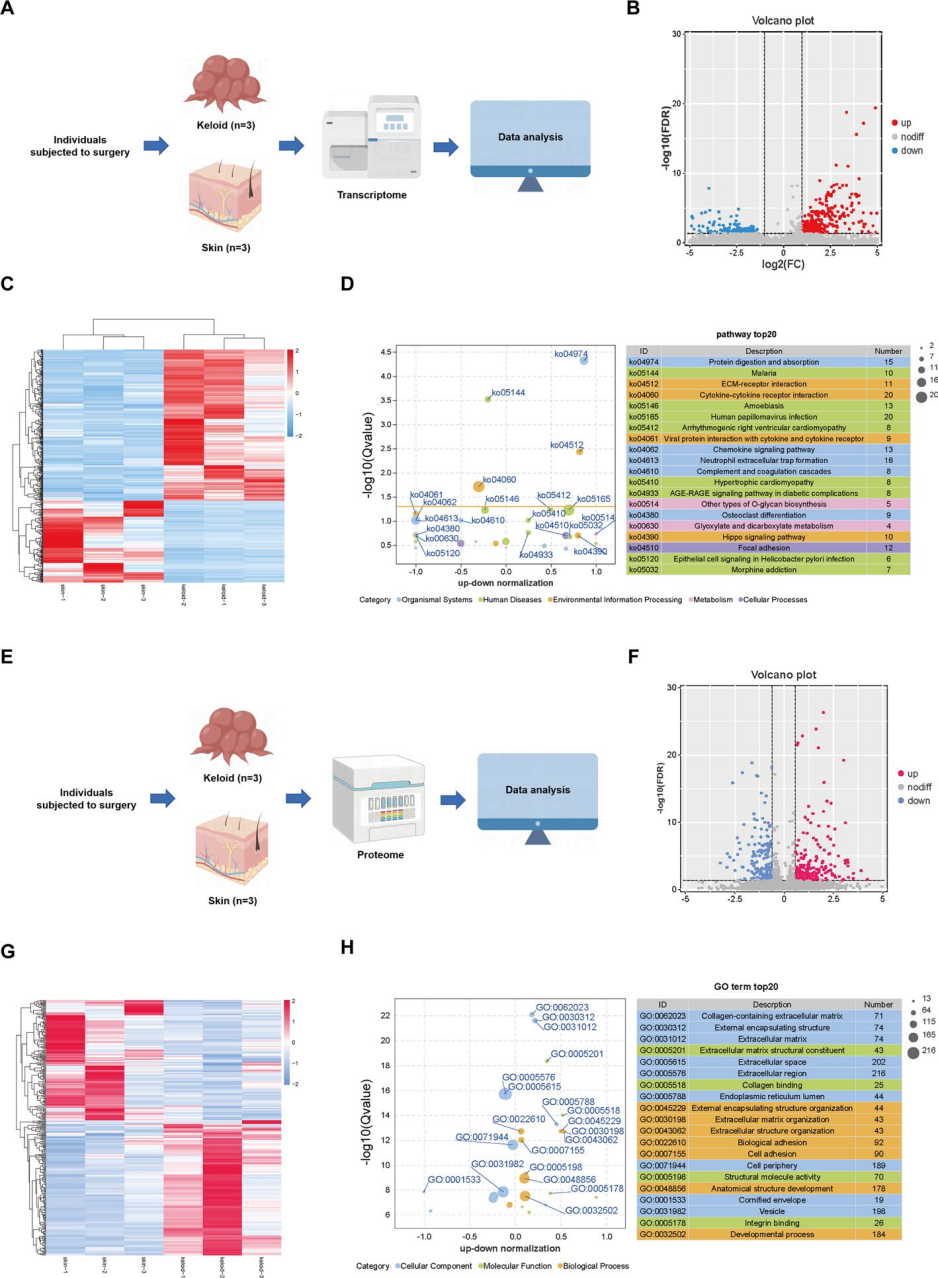
Transcriptomic and proteomic analysis of keloids. **A** Flowchart of the keloid transcriptome analysis. **B** Volcano plot of DEGs in transcription phase. **C** Heatmap of the expression of DEGs. Both rows and columns are clustered using correlation distance and average linkage. **D** KEGG pathway enrichment analysis of DEGs. Each circle represents a term, and the circle size is the number of genes that fall into the term. **E** Flowchart of the keloid proteomics analysis. **F** Volcano plot of DEGs in translation phase. **G** Heatmap of the expression of DEPs. Rows are clustered using correlation distance and average linkage. **H** GO function enrichment analysis of DEPs. Each circle represents a term, and the circle size is the number of genes that fall into the term

The libraries were specifically constructed for RNA sequencing, and deep sequencing was performed on the six samples (three normal skin tissues versus three keloid tissues) that met the quality requirements (Fig. 2A). We identified DEGs between samples using two criteria: a log fold change of |log_2_(fold change)|> 1 and a significance level of FDR-adjusted *P* < 0.05. Compared with normal skin tissue, patients with keloid showed significant differential expression of 467 genes, including 303 upregulated and 164 downregulated genes (Fig. 2B; Supplementary File 4). The clustered heatmap (Fig. 2C) demonstrates a clear separation between the control and keloid groups based on gene expression, with samples from keloid 1 and 2 exhibiting a more distinct contrast compared with controls. The KEGG enrichment analysis was conducted to investigate the signaling pathways associated with the DEGs (Fig. 2D). Keloid tissues exhibited a significant enrichment of human diseases and organismal systems that dominate the mechanism of keloid formation when compared with control tissues. Additionally, DEGs were primarily enriched in pathways related to protein digestion and absorption, focal adhesion, ECM–receptor interaction, as well as immune system activities and inflammatory response. These enriched pathways have an impact on the progression of keloid disease.

Differential analysis of proteins between three normal skin tissues and three keloid tissues was conducted on the basis of protein quantification results, aiming to identify proteins with significant changes in abundance across comparison groups (Fig. 2E). To reduce the occurrence of false positives, we utilized the Benjamini–Hochberg (BH) method for correcting multiple hypothesis testing and obtaining the FDR value. We defined DEPs as those meeting the criteria of an adjusted FDR threshold of < 0.05 and a fold change > 1.5. As a result, we identified 351 DEPs in keloid tissues, consisting of 185 upregulated proteins and 166 downregulated proteins (Fig. 2F; Supplementary File 5). As depicted in Fig. 2G, the expression levels of these proteins varied significantly across different samples, with a positive correlation between color intensity and protein abundance. GO enrichment analysis was conducted to investigate the biological process (BP), molecular function (MF), and cellular component (CC) associated with the DEPs (Fig. 2H). Figure 2H displays the top 20 enrichment outcomes across three categories, namely developmental process (ontology: BP), extracellular region (ontology: CC), and structural molecule activity (ontology: MF). These enriched pathways are closely linked to the onset and progression of keloid disease.

### Integrated omics analysis and identification of potential hub genes

**Fig. 3:**
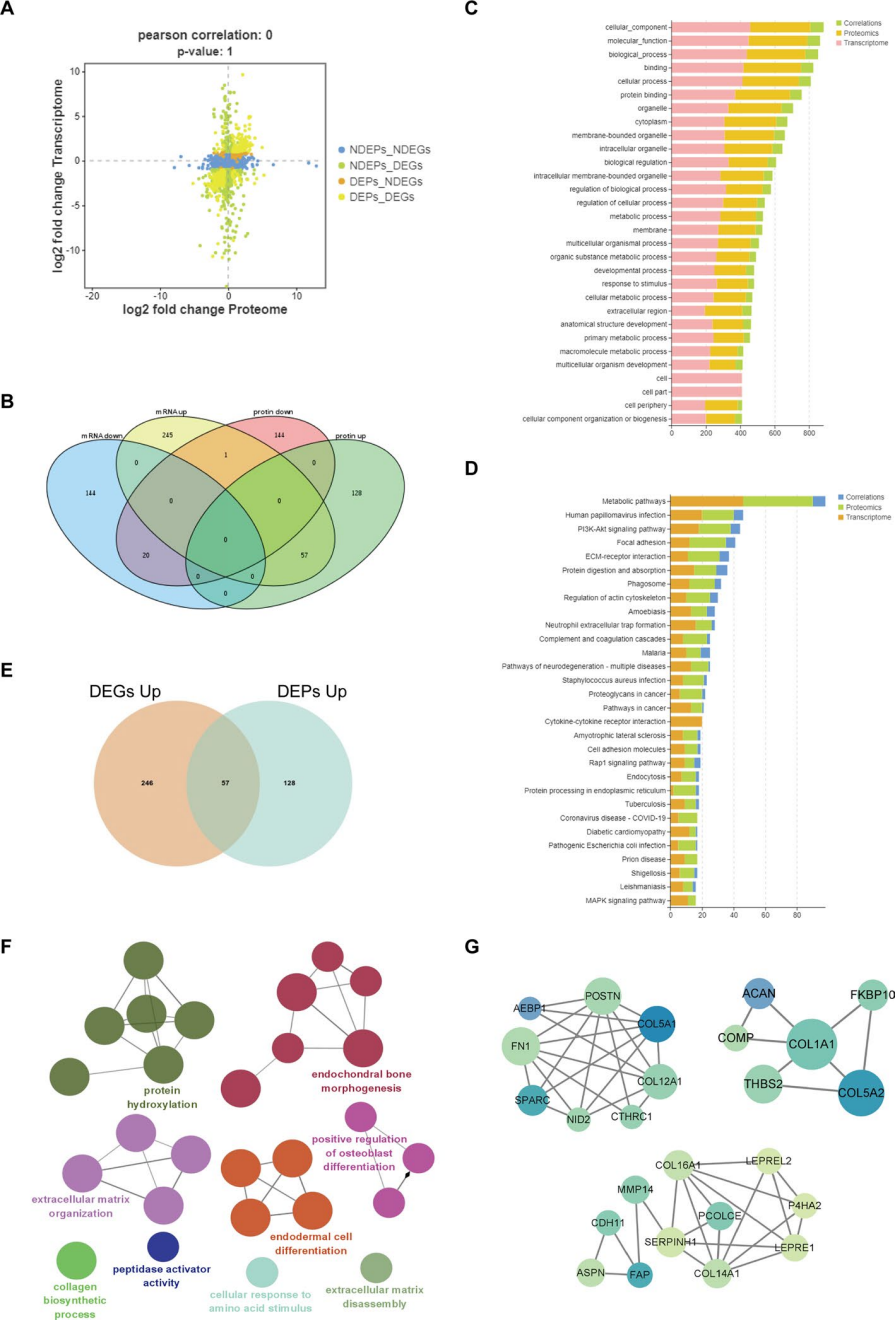
Integrated omics analysis and identification of potential hub genes. **A** Four-quadrant diagram of gene expression in transcription and translation phase. **B** Venn diagram of DEGs and DEPs. **C** Association analysis of the GO function of DEGs and DEPs. **D** Association analysis of KEGG pathway for DEGs and DEPs. **E** Venn diagram of significantly upregulated DEGs and DEPs. **F** The biological processes of overlapped 57 upregulated genes; terms are distinguished by different colors. **G** Key modules within the protein interaction network

By interactively analyzing the expression matrices of RNAs and proteins, we identified key genes that discriminate between keloid and control tissues, and investigated the correlations between mRNA and protein expression. As illustrated in Fig. 3A, the differentially expressed genes/proteins (DEPs/DEGs) were analyzed alongside non-differentially expressed genes/proteins (NDEPs/NDEGs), and subsequently divided into four modules: NDEPs–NDEGs, NDEPs–DEGs, DEPs–NDEGs, and DEPs–DEGs. It can be inferred that the DEPs–DEGs group constitutes a crucial element in the four-quadrant map. Therefore, we identified genes that exhibited significant differential expression at both transcriptional and translational stages, categorized them into four groups on the basis of their regulatory tendencies, and utilized a Venn diagram to illustrate the distribution of specific DEGs among these categories (Fig. 3B). We identified a total of 818 genes that exhibit significant differences in expression between the transcriptome and proteome. Fifty-seven were found to be significantly upregulated in both omics, while a total of 20 genes were downregulated in both omics. Interestingly, one gene exhibited a divergent regulatory pattern, being significantly upregulated during transcription but downregulated during translation. Then, we performed association analysis on the GO function and KEGG pathway information of DEGs and DEPs, comparing gene functions and metabolic pathways at two omics levels to better identify key genes or proteins in critical pathways. These DEGs and DEPs are significantly enriched in biological pathways and other high-level GO terms, playing crucial roles in cell regeneration, differentiation, and intercellular sequential generation (Fig. 3C). Through KEGG pathway analysis, it is evident that DEGs and DEPs are significantly associated with metabolism pathway, and primarily involved in regulating inflammation and intercellular communication (Fig. 3D).

On the basis of the transcriptomic and proteomic results, we aimed to further identify potential hub genes. From the DEPs and DEGs obtained in the previous step, we selected 57 genes that were significantly upregulated in both omics for subsequent investigation (Fig. 3E). To investigate the molecular and cellular mechanisms underlying keloid tissue abnormalities, we utilized Cytoscape software to annotate the biological processes of 57 upregulated genes in the GO cohort. Our analysis revealed that these genes are primarily involved in protein hydroxylation, endochondral bone morphogenesis, ECM organization, and endodermal cell differentiation (Fig. 3F). The PPI networks of the 57 upregulated genes in the keloid group were constructed using the STRING database to identify hub genes and key modules. The most significant modules were identified using the MOCDE tool in Cytoscape (Fig. 3G). The first module consisted of a network with 20 edges and 8 nodes, while the second module had a network with 21 edges and 11 nodes. The third module contained a network with only eight edges and six nodes. POSTN, COL5A1, COL12A1, SPARC, FN1, COL1A1, SERPINH1, MMP14, COL16A1, and COL14A1 were identified as significant nodes and potential hub genes owing to their strong connections with other members within the module.

### Identification and analysis of SE-associated genes in fibroblasts

**Fig. 4:**
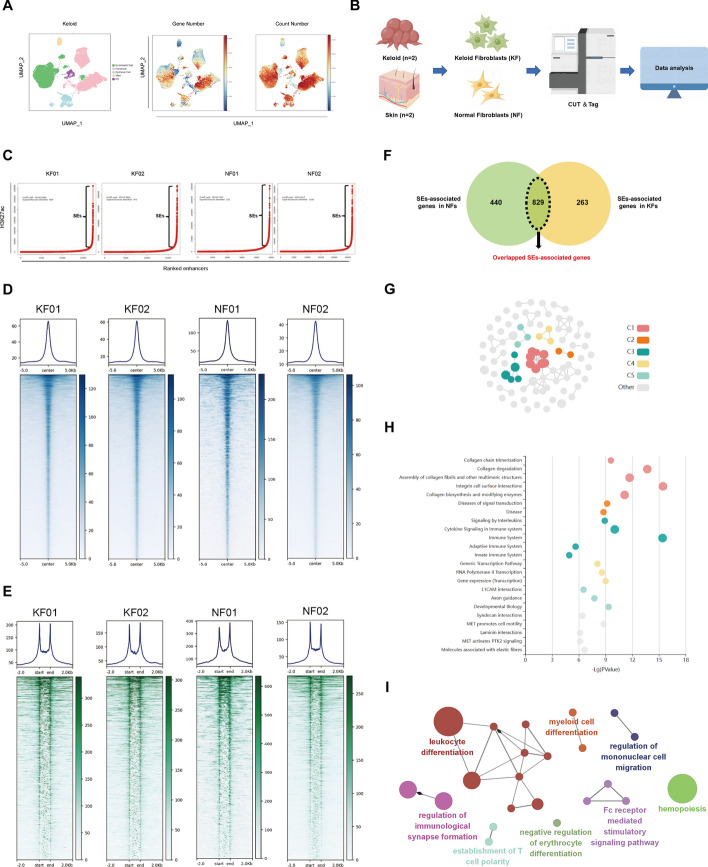
Identification and analysis of SE-associated genes in fibroblasts. **A** Dimensionality-reduction clustering of the keloid dataset (GSE163973). Clusters are distinguished by different colors, and the general identity of each cell cluster is shown on the right. Feature plots of expression distribution for gene number and count number. **B** Flowchart of the keloid super-enhancer analysis. **C** Ranked enhancer plots defined by H3K27ac. Enhancers above the inflection point of the curve have exceptionally strong H3K27ac signals and are defined as SEs. **D** Metaplots and heat map representation of H3K27A occupancy at TEs. The *x*-axis shows the center of the TE regions flanked by ±5 kb sequence. **E** Metaplots and heatmap representation of H3K27A occupancy at SEs. The *x*-axis shows the start and end of the SE regions flanked by ±2 kb sequence. **F** Venn diagram showing the intersection of SE-associated genes in keloid fibroblasts and normal fibroblasts. Overlapped SE-associated genes are marked. **G**, **H** Reactome pathway enrichment analysis of overlapped SE-associated genes using the KOBAS database. **I** GO-immunity system process analysis of overlapped SE-associated genes

To identify the crucial cellular components in keloid tissue and investigate their regulatory changes, we initially searched the GEO database for dataset GSE163973 and ensured the reliability of cell samples by conducting quality control on its single-cell data of keloid. Using UMAP clustering, five distinct cell clusters can be identified primarily (Fig. 4A). The main components are occupied by endothelial cells and fibroblasts, with the latter being more prominent in both gene number and count number (Fig. 4A). Therefore, we performed primary extraction of normal skin and keloid-derived fibroblasts, and performed cleavage under targets and tagmentation (CUT&Tag) experiments, library preparation, and computer sequencing (Fig. 4B). We utilized H3K27ac to define active enhancers across the entire genome of these cells, followed by mathematical statistical analysis to calculate enrichment intensities for each enhancer. Subsequently, we ranked all enhancers on the basis of their enrichment and identified inflection points with a slope of 1 as a threshold for distinguishing ordinary enhancers from SEs. As shown in Fig. 4C, 808 and 975 SEs were identified in keloid fibroblasts, as well as 525 and 1328 SEs in normal fibroblasts. Interestingly, our analysis of enhancers revealed that the degree of enrichment of the sequencing signals for typical enhancers (TEs) and SEs in KF were relatively lower compared with those observed in NF (Fig. 4D, E).

It is well established that SEs drive the expression of nearby genes, which are referred to as SE-associated genes. The genemapper algorithm predicts 1269 SE-associated genes in normal fibroblasts and 1092 SE-associated genes in keloid fibroblasts. By intersecting the SE-associated genes in normal and keloid fibroblasts, we identified 440 SEs that are specific to normal fibroblasts and 263 SEs that are specific to keloid fibroblasts. Considering the heterogeneity among samples, we identified common SE-associated genes in both groups for subsequent investigation. Among them, 829 SE-associated genes were found to overlap (Fig. 4F; Supplementary File 6). After conducting reactome pathway enrichment analysis using the KOBAS database, we identified five main clusters among the overlapping genes (Fig. 4G). The results of gene enrichment analysis were primarily clustered in various pathways associated with collagen and immune (Fig. 4H). Additionally, we conducted GO-immunity system process analysis to further visualize the immune biological processes associated with the overlapped SE-associated genes. The results showed that these genes mainly involve the differentiation of leukocyte and myeloid cells, regulation of immunological synapse formation and mononucleae cell migration, Fc receptor-mediated stimulatory signaling pathway, and establishment of T cell polarity (Fig. 4I).

### Screening and identification of activated SE-associated genes in keloid

**Fig. 5:**
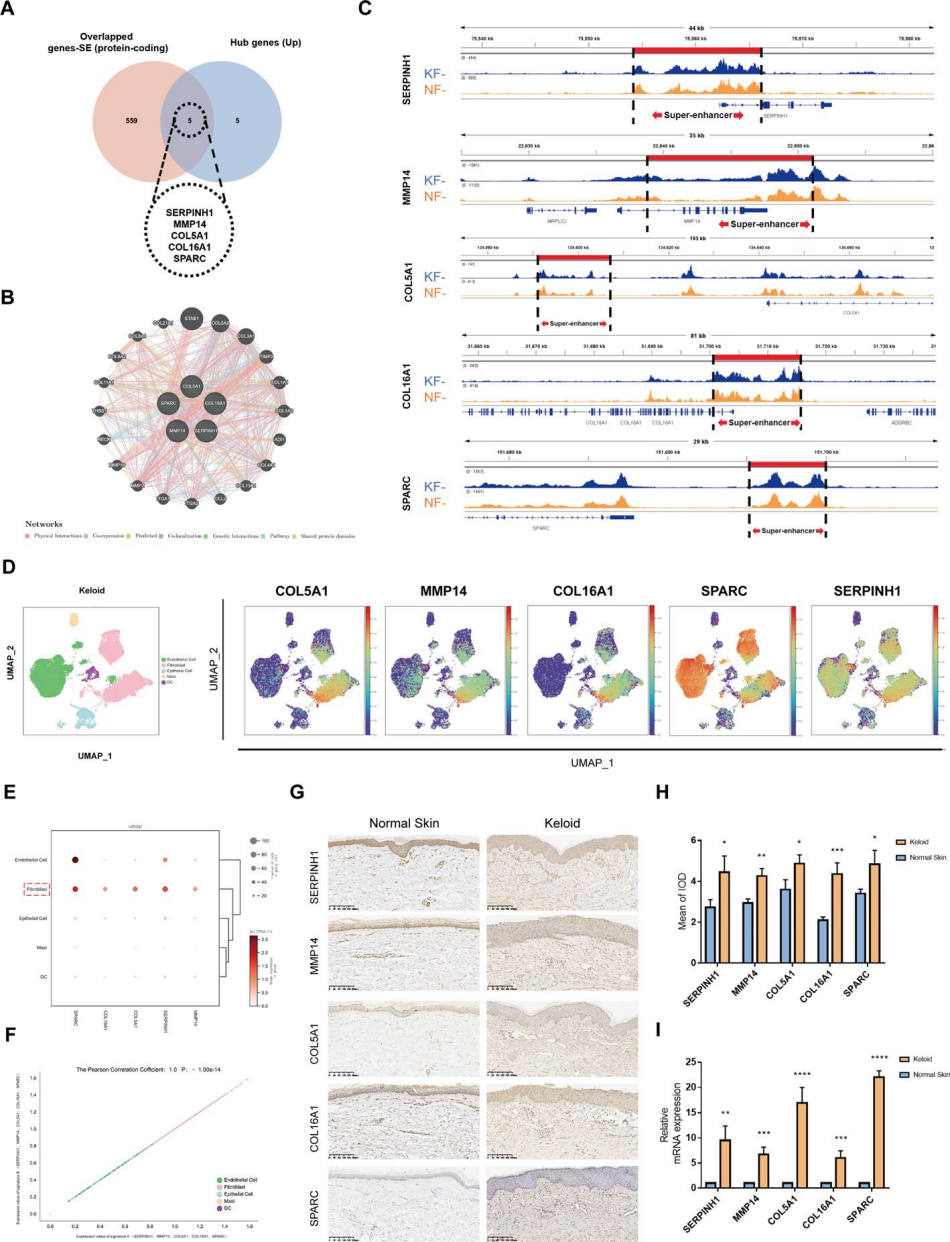
Screening and identification of activated SE-associated genes in keloids. **A** Venn diagram showing the intersection of overlapped SE-associated genes and hub genes. **B** Interaction network of five overlapped SE-associated genes and their possible related genes, constructed in the GeneMANIA database. **C** The SEs and associated genes in keloid fibroblasts and normal fibroblasts visualized using the Integrative Genomics Viewer software (version 2.10.3). **D** Feature plots of the expression distribution for five SE-associated genes in keloid. Expression levels for each cell are color-coded and overlaid onto the UMAP plot. **E** Bubble plots illustrating the expression of five SE-associated genes in each cell subpopulations. Color intensity indicates the average gene expression level. The size of the spots shows the percentage of cells expressing the gene. **F** Correlation analysis of five SE-associated genes. **G**, **H** Representative immunohistochemistry images and the mean integrated optical density (IOD) values of five SE-associated genes in keloid and normal skin tissues. **I** The relative mRNA expression levels of five SE-associated genes were measured by qPCR. *P < 0.05, **P < 0.01.***P < 0.005, ****P < 0.001

Given that SEs can significantly drive gene expression and that the overexpression of genes is a key contributor to pathogenicity, our aim was to determine which coexisting SE-associated genes in fibroblasts would be hyperactivated in keloid. These overactivated genes may represent potential pathogenic drivers in keloid development. Intersecting the overlapped protein-coding genes with previously obtained hub genes, we identified five SE-associated genes. These five genes were found in both normal and keloid fibroblasts, but exhibited overexpression only during transcription and translation stages of keloids (Fig. 5A). The GeneMANIA database visualizes the PPI network of these genes through various approaches, including physical interaction, co-expression, co-localization, pathway analysis, prediction, and genetic interactions. It is evident that the five SE-associated genes are in close proximity, and their associated genes are mainly involved in collagen synthesis and intercellular communication (Fig. 5B). The position of SEs driving the five genes (SERPINH1 SE, MMP14 SE, COL5A1 SE, COL16A1 SE, and SPARC SE) in the genome of fibroblasts are chr11:75554588–75565603, chr14:22838631–22851544, chr9:134586,172–134603217, chr1:31701072–31715669, and chr5:151696309–151699706, marked in red in Fig. 5C. We observe that there is a significant enrichment of similar H3K27ac peaks in both normal and keloid fibroblasts, which are all located proximal to their respective SEs.

Next, we first used external data for preliminary verification. Through scRNA-seq analysis, we found that these five genes (SERPINH1 SE, MMP14 SE, COL5A1 SE, COL16A1 SE, and SPARC SE) were distributed in each cell subset in keloid but mainly enriched in fibroblast subsets, while SPARC SE and SERPINH1 SE were also enriched in endothelial cells (Fig. 5D). Overall, all the five genes were significantly highly expressed in keloid fibroblasts and showed a significant positive correlation with each other (Fig. 5E and F). These genes’ mRNA transcript expression was quantified using qPCR, and their subcellular expression in tissues was investigated by IHC staining. Using staining and imaging technology, we conducted an analysis of the expression of SERPINH1 SE, MMP14 SE, COL5A1 SE, COL16A1 SE, and SPARC SE proteins in both keloid and normal skin tissues obtained from patients with keloids as well as healthy individuals (Fig. 5G). It is apparent that the epidermis and dermis of the keloid group were significantly thickened, while fibroblast-rich keloid tissue exhibited significant enrichment in high protein levels of SERPINH1 SE, MMP14 SE, COL5A1 SE, COL16A1 SE, and SPARC SE. As depicted in Fig. 5H, the protein expression levels of all five SE-associated genes were significantly elevated compared with those observed in the normal skin group. The qPCR assay also showed that the mRNA expression of SERPINH1 SE, MMP14 SE, COL5A1 SE, COL16A1 SE, and SPARC SE was significantly upregulated in keloid patients (Fig. 5I), which was basically consistent with the IHC results mentioned above.

### Prediction and analysis of functional transcription factors

**Fig. 6:**
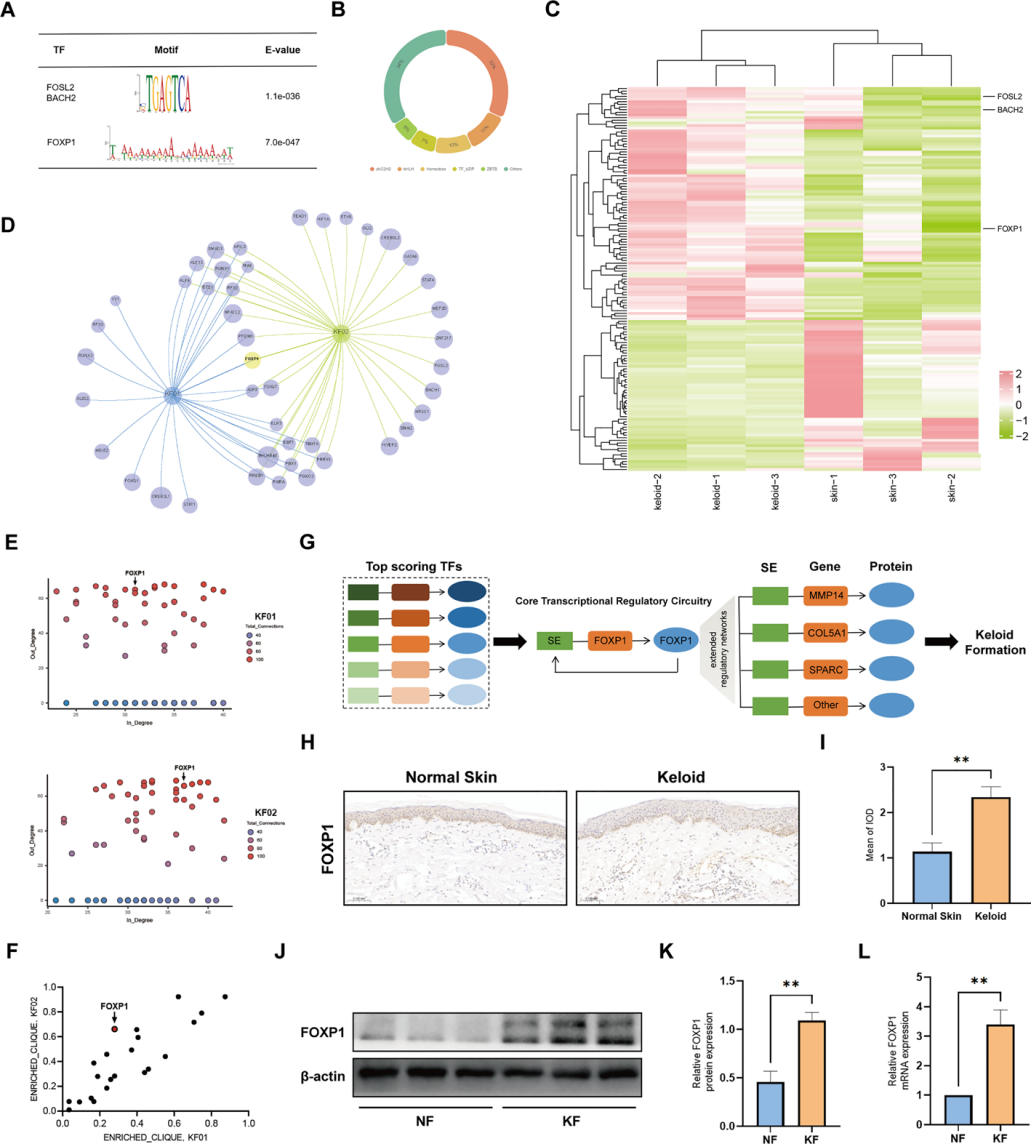
Prediction and analysis of functional transcription factors. **A** Table depicting TF binding motifs enriched at constituent enhancers within SE regions, predicted by MEME. **B** Based on the transcriptomic data of keloid and normal skin tissue to classify the significantly different transcription factors. **C** Heatmap of significantly different TFs in keloid and normal skin tissue samples. **D** Intersection plot of autoregulate TFs in different keloid fibroblasts. **E** Scatter plot depicting the relationship between in-degree (the number of transcription factors binding to a gene’s regulatory element) and out-degree (the number of regulatory elements bound by a transcription factor) for genes within the SE-mediated regulatory network. **F** The clique enrichment score for each CRC TF calculated as the proportion of total cliques in which that TF is a constituent member. **G** Schematic diagram of the FOXP1-driven core regulatory transcription factor network. **H, I** Representative immunohistochemistry images and the mean integrated optical density (IOD) values of FOXP1 in keloid and normal skin tissues. **J**, **K** The protein expression and quantitative data of FOXP1. **L** The relative mRNA expression levels of FOXP1 measured by qPCR. *P < 0.05, **P < 0.01.***P < 0.005, ****P < 0.001

SE-driven transcriptional regulation involves a sophisticated interplay between the transcriptional machinery and TFs, with a particular emphasis on identifying master regulators. Therefore, we utilized these SEs to predict their corresponding master TFs using MEME-ChIP [33]. By utilizing keloid sample data for TF analysis and target gene prediction, we identified three potential master TFs (FOXP1, FOSL2, and BACH2) that may regulate the expression of these five SE-associated genes in keloids (Fig. 6A). To clarify the expression patterns of these three TFs in keloids, we initially extracted TFs from previous transcriptomic data and conducted differential analysis. By categorizing significantly different TFs, we identified the top five categories as zf-C2H2, bHLH, Homeobox, TF_ZIP, and ZBTB. Additionally, FOSL2 and BACH2 belong to the TF_ZIP family, while FOXP1 belongs to the Fork head family (Fig. 6B). We also found that FOSL2, BACH2, and FOXP1 were significantly highly expressed in the keloid group, and the results were as shown in the heatmap (Fig. 6C; Supplementary File 7).

To further delineate the core TFs, we conducted CRC analysis and identified a total of 22 self-loop TFs in keloid fibroblasts, among which FOXP1 was selected for focused investigation (Fig. 6D). The quantification of network connectivity among TF nodes, as embodied by in-degree (the number of TFs binding to the SE of a node TF) and out-degree (the number of SEs bound by a node TF values, has revealed a group of highly connected TFs (Fig. 6E). The FOXP1 not only exhibits a high degree of connectivity but also is embedded in autoregulatory cliques with high clique enrichment fractions (Fig. 6F). Highly interconnected TFs, such as FOXP1, not only form feed-forward autoregulatory loops but also can establish an extended regulatory network (Fig. 6G). To further verify the expression of FOXP1, the IHC staining results showed that FOXP1 was significantly highly expressed in keloid tissues and could be located in the nucleus (Fig. 6H, I). The upregulation of FOXP1 mRNA and protein levels in keloid fibroblasts was further confirmed by western blot and qPCR analyses (Fig. 6J–L).

### The critical role of FOXP1 as a core transcriptional regulator

**Fig. 7:**
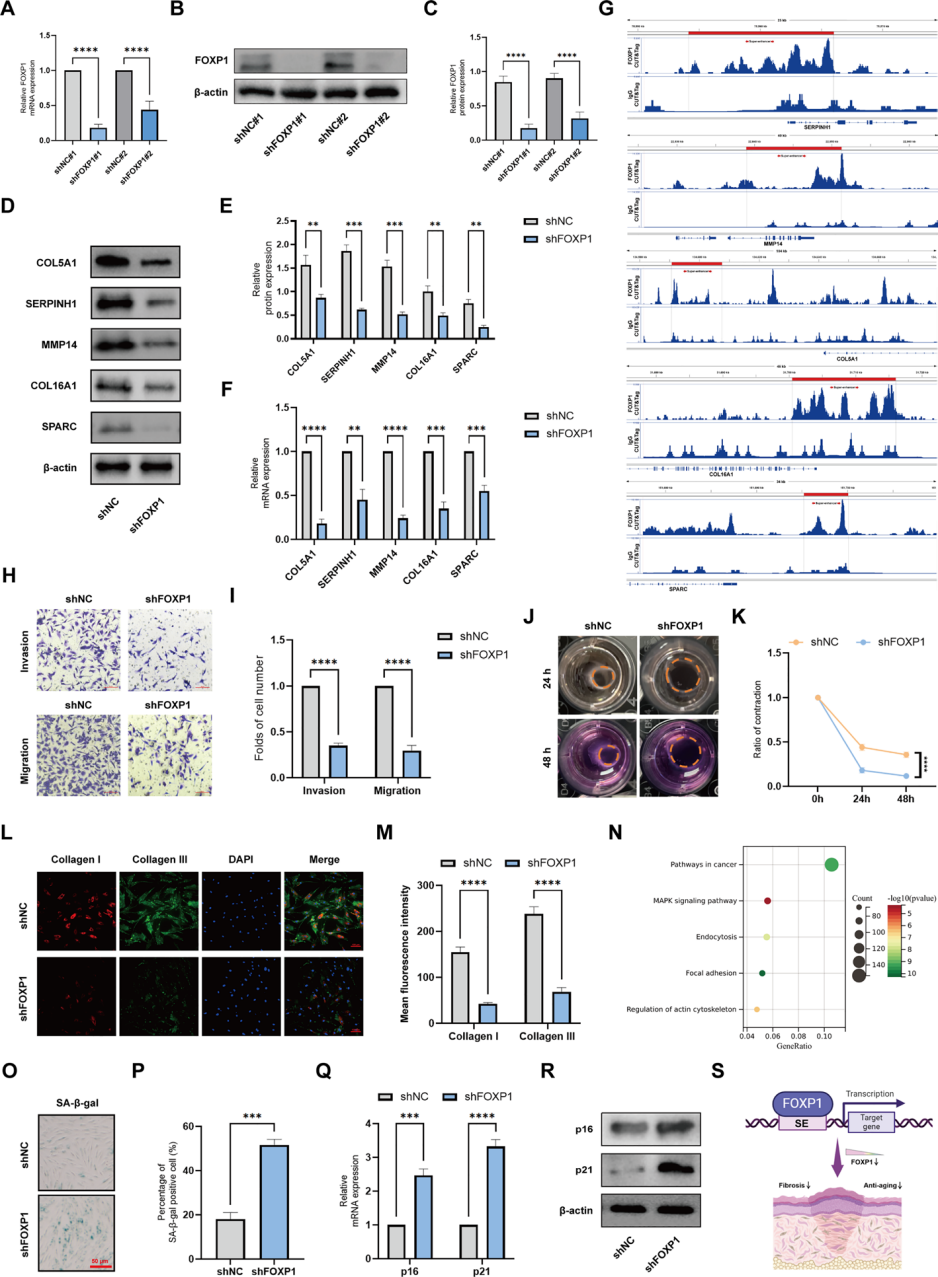
The critical role of FOXP1 as a core transcriptional regulator. **A** Western blot detecting FOXP1 protein levels after transfection of shNC and shFOXP1#1 and siFOXP1#2. **B** Quantitative data of FOXP1 protein level after transfection with shFOXP1#1 and shFOXP1#2 compared with shNC. **C** qPCR detecting silent efficacy of FOXP1 shRNA. **D, E** The protein expression and quantitative data of COL5A1, COL16A1, SPARC, MMP14, and SERPINH1. **F** The relative mRNA expression levels of COL5A1, COL16A1, SPARC, MMP14, and SERPINH1, measured by qPCR. **G** CUT&Tag identified binding site of FOXP1 in SE regions of COL5A1, COL16A1, SPARC, MMP14, and SERPINH1. **H**, **I** Representative images and statistics of cell migration and invasion assays. **J, K** Representative images and statistics of collagen gel contraction assay. **L** Representative images of keloid fibroblasts stained with collagen I and collagen III. **M** Analysis of fluorescence density of collagen I and collagen III. **N** Representative KEGG pathways of FOXP1 target genes identified by FOXP1-CUT&Tag. **O**, **P** Representative images and statistics of SA-β-gal staining. **Q** qPCR detecting the senescence marker p16 and p21. **R** The protein expression of p16 and p21. **S** Schematic illustration showing that downregulation of FOXP1 can inhibit anti-senescence effects and fibrosis progression. *P < 0.05, **P < 0.01.***P < 0.005, ****P < 0.001

To confirm the relationship between FOXP1 and SE-associated genes in keloid, we knocked down FOXP1 with shRNAs in keloid fibroblasts. After shRNA transfection, we validated FOXP1 reduction at both RNA and protein expression levels by qPCR and western blot (Fig. 7A–C). Meanwhile, we found that knockdown of FOXP1 reduced the mRNA and protein expression levels of SERPINH1 SE, MMP14 SE, COL5A1 SE, COL16A1 SE, and SPARC SE (Fig. 7D–F). The results of the FOXP1-Cut&Tag experiment in keloid fibroblasts revealed that FOXP1 is associated with the SEs of SERPINH1, MMP14, COL5A1, COL16A1, and SPARC, all of which exhibited significant signal enrichment in their SE regions (Fig. 7G). Collectively, these findings suggest that FOXP1 plays a crucial role in SE-driven molecular changes through its binding activity.

Because the function of FOXP1 in chronic fibrosis of the skin is unknown, we performed the relevant phenotype identification to investigate the effect of FOXP1 on keloid formation. On the basis of cell migration and invasion assays, we observed that knockdown of FOXP1 significantly suppresses the migration and invasion capabilities of keloid fibroblasts (Fig. 7H, I). Additionally, downregulation of FOXP1 markedly inhibits the gelatin contraction ability of keloid fibroblasts (Fig. 7J, K). To further validate the importance of FOXP1 in collagen formation, immunofluorescence results demonstrate that FOXP1 knockdown significantly reduces the synthesis levels of type I and type III collagens (Fig. 7L, M). According to target gene enrichment analysis of FOXP1 in keloid fibroblasts, FOXP1 primarily regulates pathways related to cancer and the MAPK signaling pathway (Fig. 7N; Supplementary File 8). Previous literature suggests that FOXP1 can promote cancer progression and participates in the senescence processes of various organ tissues [34, 35]. The enriched MAPK signaling is crucial for regulating cellular senescence [36]. To explore whether FOXP1 mediates senescence regulation in fibrosis, we found that knockdown of FOXP1 in keloid fibroblasts simultaneously resulted in accelerated senescence, as characterized by increased SA-β-gal activity and increased expression of the senescence markers p16 and p21 (Fig. 7O–R). In conclusion, our findings highlight FOXP1 as a core TF that orchestrates the expression of downstream genes. The downregulation of FOXP1 not only suppresses anti-senescence pathways but also mitigates fibrotic processes, offering new insights into the mechanisms for treating skin fibrotic diseases (Fig. 7S).

## Discussion

Keloid formation is a complex process involving various cell types, inflammatory and immune responses, as well as growth factors [37]. Recent advances in multi-omics analysis have provided deeper insights into disease pathogenesis, but research on keloids has predominantly focused on transcriptomics, with limited studies on other omics. In this study, we integrated transcriptomic and proteomic analyses of keloid tissue to describe the expression characteristics and gene expression-related networks in patients with keloid.

Our findings revealed that DEGs in patients with keloid were enriched in pathways related to the ECM and immune responses. Similarly, DEPs were concentrated in pathways associated with ECM production and deposition. This suggests that ECM is a crucial determinant in keloid development, and alterations in immune-related gene transcription may lead to increased ECM production, consistent with previous research [38]. Keloids have been closely associated with persistent inflammatory responses, including the infiltration of inflammatory cells, which trigger fibroblast proliferation and excessive ECM deposition, resulting in keloid formation [39, 40].

Through integrative omics analysis, we observed both consistent and inconsistent changes in mRNA and protein expression. Besides the strong correlation between ECM and immune-related pathways, DEGs and DEPs were also significantly associated with metabolic pathways, highlighting metabolism as a critical focus in keloid research. Some studies have observed metabolic disorders in keloids, including reprogramming from oxidative phosphorylation to aerobic glycolysis [41, 42]. Keloid fibroblasts may also play an important role in metabolic variation [43–45]. We conducted further investigations on the genes that exhibited significant differences between these two stages and identified novel biomarkers for keloids. We identified 57 genes that were significantly upregulated during both transcription and translation. These genes were predominantly involved in protein post-translational modifications, ECM remodeling, and other key processes in tissue growth, particularly within mesenchymal stem cells and fibroblasts in the keloid microenvironment [46–48]. Among these, 10 hub genes were identified through modular analysis, suggesting their potential importance in keloid pathogenesis and progression.

Keloid fibroblasts, as key effectors in fibrosis, play a central role in keloid progression. Differences in gene expression between transcriptional and translational stages highlight the regulatory role of fibroblasts in keloids. Using single-cell data, we confirmed the significant involvement of fibroblasts in keloid tissue. SEs, which recruit numerous TFs and cofactors to induce target gene transcription, have been linked to cancer genes [14, 49, 50]. Although their role in benign tumors such as keloids is unclear, our study identified 829 SE-associated genes in both keloid and normal skin fibroblasts. Enrichment analysis results indicate that these genes are also linked to collagen and immunity. To further elucidate the specific genes activated in the keloid microenvironment, we performed an intersection analysis of these 829 genes with 10 previously identified hub genes that exhibit significant upregulation during transcription and translation stages in keloids. Five SE-associated genes (SERPINH1 SE, MMP14 SE, COL5A1 SE, COL16A1 SE, and SPARC SE) were significantly upregulated in keloids, particularly in fibroblasts, as confirmed by single-cell sequencing data, qPCR, and IHC analyses.

Given the potential of SE-regulated proteins as drug targets, we explored their roles further. SERPINH1 functions as a molecular chaperone specific to collagen, briefly interacting with procollagen during folding, assembly, and transport within the endoplasmic reticulum [51]. The inhibition of SERPINH1 effectively suppresses collagen secretion, leading to a notable amelioration of liver fibrosis in mice [52]. As a constituent of the ECM, SPARC plays a pivotal role in numerous biological processes [53]. Emerging evidence has demonstrated that SPARC promotes excessive ECM protein deposition in idiopathic pulmonary fibrosis and keloid, as well as influences the proliferation and migration of fibroblasts [54–56]. Besides, research shows that SPARC is able to convert anti-inflammatory macrophages into a pro-inflammatory phenotype, and deletion of SPARC may protect against high-fat diet-induced chronic inflammation by preventing activation of the NLRP3 inflammasome [57, 58]. As a catabolic factor, MMP14 has the capacity to directly degrade ECM components [59, 60]. Studies of fibrotic organ lesions prove that overexpression of MMP14 can drive inflammation [61–63]. Additionally, this enzyme is involved in various biological processes, including proliferation, invasion, angiogenesis, and basement membrane remodeling [64, 65]. As a minor constituent of fibrogenic collagen, type V collagen plays a crucial role in the organization of the ECM. It typically forms copolymers with type I or II collagen to regulate the length and abundance of heterotypic collagen fibrils [66]. Collagen XVI is a minor component of the connective tissue that regulates ECM integrity and stability, while also serving as a substrate for tumor cell adhesion and invasion to alter cell–matrix interactions and induce invasion [67]. In conclusion, these five SE-associated genes may serve as crucial influencing factors in the pathogenesis of keloid hyperfibrosis. In the context of chronic inflammation promoting fibrosis [68], the studied genes’ association with inflammation requires further research. 

Targeting SEs and their downstream genes could offer new therapeutic strategies for keloids. The previous reports have not mentioned or clarified the potential therapeutic targets for keloid of these five SE-associated genes in relation to SEs. However, pharmacological intervention has shown particular sensitivity toward SEs, thus presenting great potential as a therapeutic target by controlling SE-activated gene transcription and protein translation [14]. Various inhibitors targeting SEs have been developed, such as BRD4 inhibitors, histone acetylation inhibitors, and CDK inhibitors. Additionally, targeting SE complexes and utilizing gene editing techniques have shown promise as effective strategies [69, 70]. Owing to the limitations of this study, we were unable to establish a specific causal relationship between these interaction variables. Future studies utilizing new experimental designs are necessary for further investigation. Additionally, SEs can serve as a prognostic marker for predicting disease risk and progression. Combining gene transcriptional signatures with SE profiles in patients or healthy individuals can provide valuable insights into disease diagnosis. We emphasize the crucial role of aberrant SE generation in keloid pathogenesis and explore the potential of targeting SEs and their downstream genes as therapeutic strategies and biomarkers for disease progression assessment.

SEs regulate gene expression through high-density epigenetic modifications that are closely associated with TFs and cofactors [71]. SEs are occupied by master TFs [13]. These TFs can aggregate at the enhancers of numerous active cell-type-specific genes, thereby contributing to the organization of gene expression patterns in that particular cell type [72]. SEs differ from typical enhancers in terms of their size, density and content of TFs, ability to activate transcription, and sensitivity to perturbation [73]. Moreover, genes associated with SEs exhibit heightened sensitivity to diminished levels of enhancer binding factors and cofactors, while the activity of SEs is more susceptible to TF blockade than that of typical enhancers [17]. The intimate association between transcriptional dysregulation and cancer evolution is underscored by the fact that numerous oncogenes and tumor suppressor genes encode TFs, strongly suggesting that aberrant gene regulation represents a fundamental mechanism underlying cancer progression [17]. By predicting the major TFs upstream of the identified SE-associated genes, it was discovered that FOSL2, BACH2, and FOXP1 were not only highly enriched in keloid fibroblasts but also significantly upregulated in keloids. Therefore, they are considered to be the master TFs targeting and regulating these SE-associated genes. Although no studies have been conducted on the involvement of FOXP1, FOSL2, and BACH2 in keloids, bioinformatics analysis of fibroblast transcriptome in systemic sclerosis (SSc) has revealed that upstream TFs (FOSL2 and FOXP1) drive myofibroblast differentiation and regulate collagen production; BACH2 has also been reported to be involved in the skin fibroblast photoaging process [74, 75]. To further investigate the mechanism of TFs, we conducted CRC analysis as an additional approach. The core TFs form an interconnected autoregulatory loop known as the CRC. Within this circuitry, TFs engage in self-regulation by binding to their own SEs, as well as to the SEs of other TFs [31]. Recent research has indicated that the CRC model plays a pivotal role in tissue development, maintenance of homeostasis, and progression of diseases [76, 77]. On the basis of the results of CRC analysis, FOXP1 was further identified as a core TF and was specifically upregulated in keloid and keloid fibroblasts. In vitro studies revealed that FOXP1 binds to the SE regions of target genes, playing a regulatory role in their expression.

Epigenetic alterations are increasingly recognized as key drivers of the progression of fibrotic diseases. In these diseases, the expression of epigenetic regulators becomes deregulated, leading to changes in the epigenetic code that favor the activation of profibrotic gene expression profiles [78]. Consequently, effector cells such as fibroblasts are able to maintain their activated phenotype even in the absence of external stimuli. FOXP1, a transcriptional regulator, has been found to be intricately involved in modulating gene expression profiles that are critical for maintaining cellular homeostasis [34, 35]. However, in the context of fibrosis, the dysregulation of FOXP1 expression may contribute to the aberrant activation of fibroblasts. Our in vitro experiments have demonstrated the ability of FOXP1 knockdown to inhibit the migration and invasion of keloid fibroblasts and collagen formation. Additionally, we have found that knocking down FOXP1 mediates the onset of cellular senescence. Cellular senescence, a current research hotspot in skin fibrosis, has been shown to play a crucial role in this process. Studies suggest that defects in stress-induced premature senescence (SIPS) can lead to an insufficient number of senescent cells, weakening their inhibitory effects and resulting in uncontrolled fibroblast proliferation, which contributes to excessive skin fibrosis [79]. Similarly, when fibroblasts undergo senescence, it further impacts cell proliferation and ECM synthesis, thereby reducing the occurrence of excessive skin fibrosis [80]. FOXP1 plays a pivotal role in the regulation of non-senescent fibrosis, particularly in the context of keloid formation. As a TF, FOXP1 is intricately involved in the activation of SEs that drive the expression of key genes associated with fibrosis. In keloid tissues, the elevated expression of FOXP1 contributes to the persistence and proliferation of fibroblasts, bypassing cellular senescence mechanisms that typically limit tissue overgrowth. This non-senescent state, maintained by FOXP1, promotes the excessive deposition of ECM proteins, leading to the characteristic thickening and scarring observed in keloids. Understanding the role of FOXP1 in this process provides valuable insights into potential therapeutic targets for treating fibrotic skin diseases, where inhibiting FOXP1 could help restore normal cellular senescence and reduce pathological scarring.

We already know that keloids have a preference to occur at sites subject to mechanical forces, such as the chest and upper arms. The specific shapes of keloids, namely crab’s claw, or dumbbell shapes, indicate that the dysregulation of mechanical transduction pathways promotes keloid formation [81]. The mechanical signaling pathways involved in the formation and growth of skin fibrosis include TGF-β/Smad, YAP/TAZ/TEAD, and so on [82, 83]. Downregulation of KLF2 expression caused by oscillatory shear stress can lead to reduction of FOXP1 and atherogenesis [84]. In this context, we propose that mechanical stress may be the potential trigger for FOXP1. We plan to verify the upstream regulators of FOXP1 in subsequent work to further refine the understanding of FOXP1 in keloids and other fibrous diseases.

Previous studies have shown that gene expression in fibroblasts is regulated by a triplet coordinate system of anatomical location: anterior–posterior, proximal–distal, and dermal–nondermal [85]. Although some researches have reported that scar type is more likely to be the core factor driving gene differential expression and pathological mechanism than anatomical location [86], the problem of confounding factors that do not match the anatomical location remains owing to the limited clinical source of samples. In the future, we plan to expand the sample size to cover multisite pairs and combine spatial transcriptome technology to further verify the current results and analyze the effects of local microenvironment on gene expression in fibroblasts.

## Conclusions

In this study, we conducted a comprehensive analysis of gene expression in keloid patients using transcriptome and proteome sequencing, as well as CUT&Tag technology. Our findings revealed five potential biomarkers—SERPINH1 SE, MMP14 SE, COL5A1 SE, COL16A1 SE, and SPARC SE. Additionally, we discovered that FOXP1 functions as a core TF promoting fibrosis and anti-senescence during keloid progression. The dual role of FOXP1 suggests its therapeutic potential as a target for managing fibrotic diseases and offers novel avenues for modulating these epigenetic mechanisms.

## Supplementary Information

Additional file 1.

Additional file 2.

Additional file 3.

Additional file 4.

Additional file 5.

Additional file 6.

Additional file 7.

Additional file 8.

Additional file 9.

## Data Availability

Data used to support the findings of this study are available from the corresponding author upon reasonable request.

## Abbreviations

SEs: Super-enhancers
TFs: Transcription factors
CRC: Core transcription regulatory circuitry
ECM: Extracellular matrix
DEGs: Differentially expressed genes
DEPs: Differentially expressed proteins
KEGG: Kyoto Encyclopedia of Genes and Genomes
GO: Gene Ontology
PPI: Protein–protein interaction
MCODE: Molecular complex detection
scRNA-seq: Single-cell RNA sequencing
UMAP: Uniform manifold approximation and projection
CUT&Tag: Cleavage under targets and tagmentation
RT-qPCR: Real-time quantitative reverse transcription PCR
IHC: Immunohistochemical staining
SA-β-gal: Senescence-associated β-galactosidase
IF: Immunofluorescence
SEM: Standard error of the mean
BP: Biological process
MF: Molecular function
CC: Cellular component
NDEPs: Non-differentially expressed proteins
NDEGs: Non-differentially expressed genes
TEs: Typical enhancers
