# Supplementary material for: Comprehensive analysis of keloid super-enhancer networks reveals FOXP1-mediated anti-senescence mechanisms in fibrosis
Source: Cell Mol Biol Lett. 2025 Jul 23;30:88. doi: 10.1186/s11658-025-00763-1 (PMC12288304; doi:10.1186/s11658-025-00763-1)
Supplement: Supplementary file 2 — Additional file 2. [file 11658_2025_763_MOESM2_ESM.docx]

**Supplementary File 2: Data of sequences for qPCR**

| **Gene** | **Sequence 5’-3’** |
| --- | --- |
| COL5A1 | Forward: TACAACGAGCAGGGTATCCAG  Reverse: ACTTGCCATCTGACAGGTTGA |
| SERPINH1 | Forward: TGCTAGTCAACGCCATGTTCT  Reverse: ATAGGACCGAGTCACCATGAA |
| MMP14 | Forward: CGAGGTGCCCTATGCCTAC  Reverse: CTCGGCAGAGTCAAAGTGG |
| COL16A1 | Forward: CCACCAGAAGACGTGGTATCT  Reverse: CAGGACACAAAGTCGCCATC |
| SPARC | Forward: CCCATTGGCGAGTTTGAGAAG  Reverse: CAAGGCCCGATGTAGTCCA |
| GAPDH | Forward: ACAACTTTGGTATCGTGGAAGG  Reverse: GCCATCACGCCACAGTTTC |
| P16 | Forward: ATGGAGCCTTCGGCTGACT  Reverse:GTAACTATTCGGTGCGTTGGG |
| P21 | Forward: CGATGGAACTTCGACTTTGTCA  Reverse: GCACAAGGGTACAAGACAGTG |
