# Supplementary figures and images for: Comprehensive analysis of keloid super-enhancer networks reveals FOXP1-mediated anti-senescence mechanisms in fibrosis
Source: Cell Mol Biol Lett. 2025 Jul 23;30:88. doi: 10.1186/s11658-025-00763-1 (PMC12288304; doi:10.1186/s11658-025-00763-1)

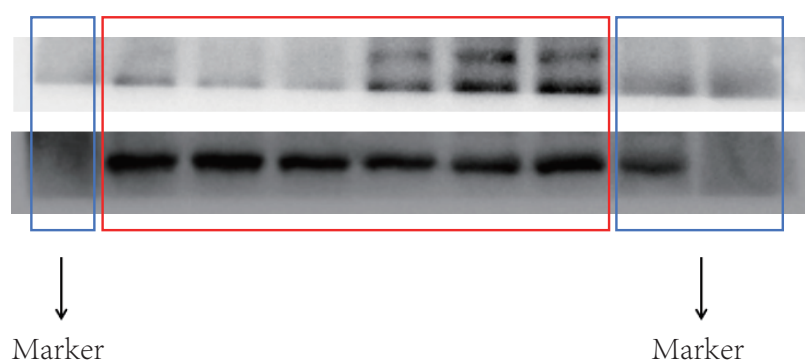

J

Figure 6

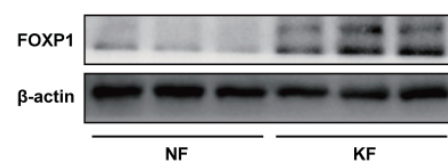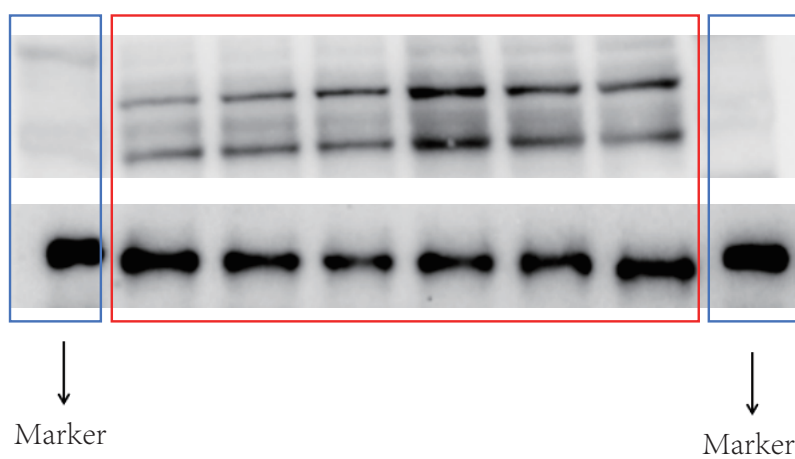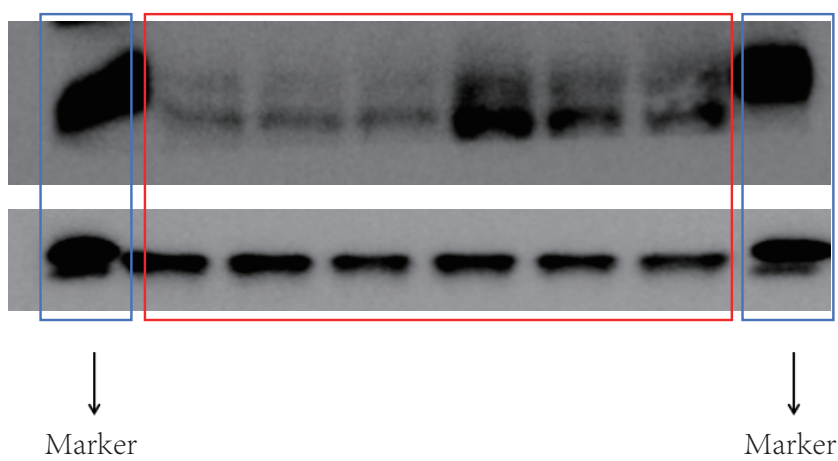

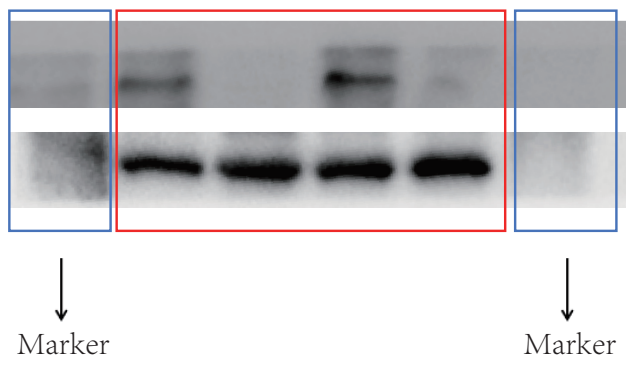

**B**

Figure 7

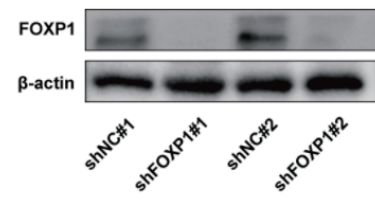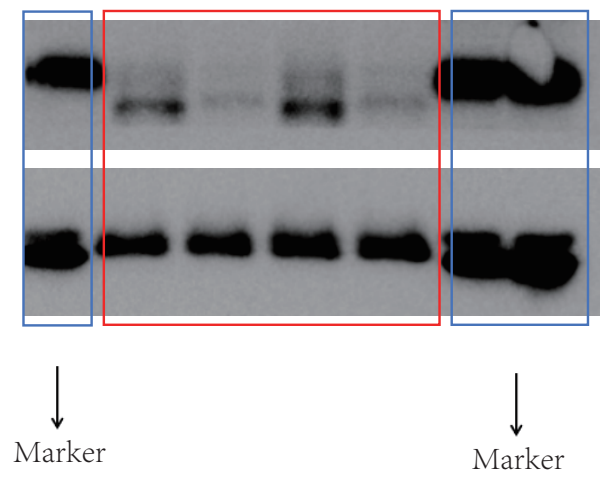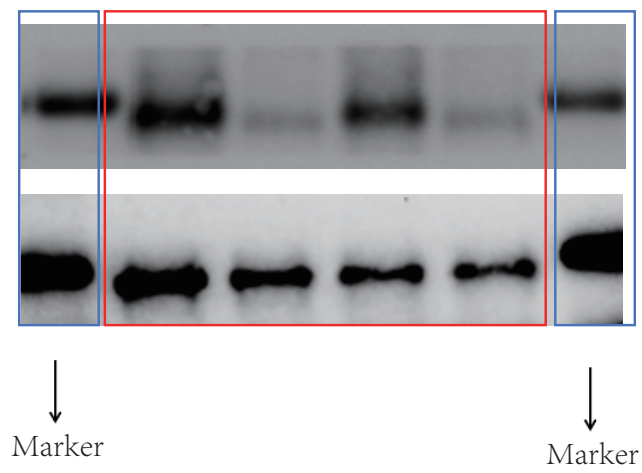

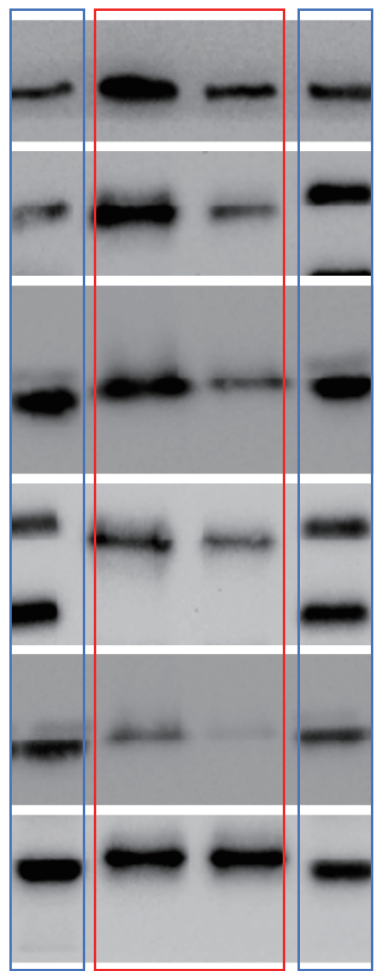

↓  
Marker

↓  
Marker

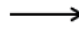

**D**

Figure 7

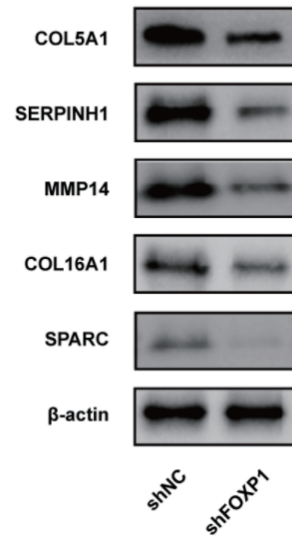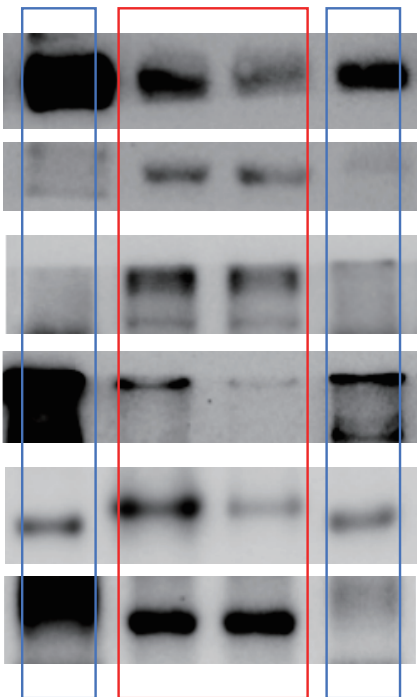

↓  
Marker

↓  
Marker

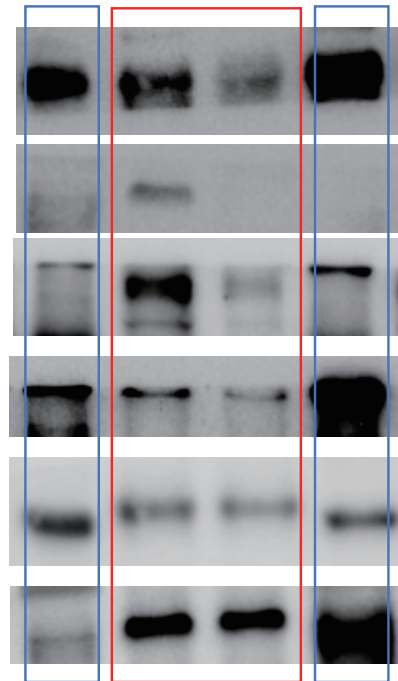

↓  
Marker

↓  
Marker

Supplement: Supplementary file 9 — Additional file 9. [file 11658_2025_763_MOESM9_ESM.pdf]
